# Thymus Functionality Needs More Than a Few TECs

**DOI:** 10.3389/fimmu.2022.864777

**Published:** 2022-06-10

**Authors:** Pratibha Bhalla, Dong-Ming Su, Nicolai S. C. van Oers

**Affiliations:** ^1^ Department of Immunology, The University of Texas Southwestern Medical Center, Dallas, TX, United States; ^2^ Department of Microbiology, Immunology & Genetics, The University of North Texas Health Sciences Center, Fort Worth, TX, United States; ^3^ Department of Microbiology, The University of Texas Southwestern Medical Center, Dallas, TX, United States; ^4^ Department of Pediatrics, The University of Texas Southwestern Medical Center, Dallas, TX, United States

**Keywords:** thymus, *FOXN1*, thymus epithelial cells, mesenchymal cells, endothelial cells, T cell development, thymus regeneration, thymus organoid technologies

## Abstract

The thymus, a primary lymphoid organ, produces the T cells of the immune system. Originating from the 3^rd^ pharyngeal pouch during embryogenesis, this organ functions throughout life. Yet, thymopoiesis can be transiently or permanently damaged contingent on the types of systemic stresses encountered. The thymus also undergoes a functional decline during aging, resulting in a progressive reduction in naïve T cell output. This atrophy is evidenced by a deteriorating thymic microenvironment, including, but not limited, epithelial-to-mesenchymal transitions, fibrosis and adipogenesis. An exploration of cellular changes in the thymus at various stages of life, including mouse models of in-born errors of immunity and with single cell RNA sequencing, is revealing an expanding number of distinct cell types influencing thymus functions. The thymus microenvironment, established through interactions between immature and mature thymocytes with thymus epithelial cells (TEC), is well known. Less well appreciated are the contributions of neural crest cell-derived mesenchymal cells, endothelial cells, diverse hematopoietic cell populations, adipocytes, and fibroblasts in the thymic microenvironment. In the current review, we will explore the contributions of the many stromal cell types participating in the formation, expansion, and contraction of the thymus under normal and pathophysiological processes. Such information will better inform approaches for restoring thymus functionality, including thymus organoid technologies, beneficial when an individuals’ own tissue is congenitally, clinically, or accidentally rendered non-functional.

## Introduction

The thymus originates from the 3^rd^ pharyngeal pouches (3^rd^ PP), budding from one of the 5 temporary bilateral evaginations developing along the embryonic gut tube [reviewed in (1, 2)]. At this early stage, *Paired Box 1* (*PAX1*), a member of the *PAX* family of transcription factors, is required for the patterning of these pharyngeal pouches. *PAX1* is present in mesenchymal condensates surrounding the pouches, including the 3^rd^ PP. Mutations in *PAX1* cause thymus hypoplasia/aplasia, a phenotype more penetrant in humans than mice (3–6). The 3^rd^ PP first patterns into the anterior-dorsal parathyroids at embryonic days 9.5-10 in the mouse, a time frame matching weeks 6-7 of gestation in humans. The ventrally positioned thymus anlage is specified at mouse embryonic days 10.5-11 (week 8 in human) (2). Both the parathyroids and thymus pattern as neural crest cell derived (NCC) mesenchymal cells (Mes) condense around a layer of columnar epithelial cells. Mes release Bone Morphogenic Protein 4 (*BMP4*), supporting the differentiation of some endothelial cells into thymus epithelial cells (TECs) (**Table 1**) (7). Vascular endothelial growth factor (VEGF), produced by Mes, and to some extent, by immature TECs and CD4^-^CD8^-^ thymocytes, aids in tissue vascularization (8–11). A major change in the thymus occurs at e11.25 as immature TECs begin expressing the *Forkhead Box N1* (*FOXN1*) transcription factor (1, 12, 13). FOXN1 positively regulates the expression of hundreds of genes, creating an environment suitable for T cell development (14). Among the up-regulated genes are those encoding chemokines, such as CCL25, which recruit early thymus progenitors (ETPs; also referred to as thymus progenitor cells) into the thymus tissue (15). The ETPs enter prior to tissue vascularization (16, 17). Thereafter, non-hematopoietic lineage cells, Mes, TECs and endothelial cells collectively cross-communicate with the hematopoietic cells to create a highly branched, three-dimensional (3D) epithelial meshwork (9, 18–21). This contrasts almost all other epithelial tissues in the body where cuboidal, squamous, or columnar features are retained (20, 22). As the thymus expands, Mes cells differentiate, forming the capsule, septae, perivascular cells (pericytes), vascular smooth muscle cells and fibroblasts. Pericytes envelope the endothelial vasculature and aid in nascent blood vessel formation (23). At early embryonic stages of thymopoiesis, T cell development proceeds in waves, with the earliest cell populations representing the γδ T cell lineage. Step-wise hematopoietic/non-hematopoietic cell-cell interactions promote thymus tissue expansion (19). These events are regulated by chemokine gradients, growth factor- and TNF- signaling pathways and Notch-notch ligand interactions, enabling immature TEC differentiation into two major subsets, cortical (cTECs) and medullary (mTECs) (**Table 1**). Cortical TECs secrete IL-7 and produce Delta-Ligand Like 4 (DLL4), supporting the expansion and differentiation of immature CD4^-^CD8^-^ (DN) thymocytes (24–26). DLL4-Notch interactions induce expression of recombination activating genes (RAG1 and RAG2), initiating V(D)J recombination at the TCR beta locus (27). Those DN thymocytes successfully expressing the TCR beta protein signal *via* the pre-TCR complex, expand, and differentiate into CD4^+^CD8^+^ (DP) cells. At the DP stage, the TCR alpha locus undergoes RAG-mediated VJ recombination. DP cells successfully forming a cell surface αβ TCR complex then undergo a maturation/selection process. In this process, thymocytes expressing the appropriate TCR undergo positive and negative selection (28, 29). The selection is dictated by the capacity of the TCR to recognize self-peptide/MHC complexes expressed on the surface of either cTECs (mainly inducing positive selection) or mTECs (primarily inducing negative selection) [reviewed in (29–31)]. Positive selection establishes TCR self-restriction, which refers to those T cells with TCRs that have a weak avidity for self-peptide-self-MHC molecules. The selected thymocytes expand and differentiate into either CD4 or CD8 single positive (SP) cells. Countering positive selection, T cells expressing a TCR with too high an affinity for self-peptide/MHC are purged from the pool of immature thymocytes, a process termed negative selection. While negative selection can occur at the both DP and SP stages (29), a more robust clearance of potentially autoreactive T cells occurs when the SP cells enter the medullary region of the thymus (32). Therein, autoimmune regulator (AIRE) expressing mTECs purge autoreactive T cells [reviewed in (33, 34)]. The negative selection process also relies on thymus dendritic cells, which present peptide-MHC complexes stripped from the mTECs (35, 36). Finally, mTECs support the development of T regulatory cells (Tregs), a subset of CD4 SP cells that control the autoreactive potential of mature T cells due to their inherent ability to recognize self-peptide/MHC (30, 32, 37). Mature SP thymocytes surviving the selection gauntlet leave the thymus. This again involves chemokine gradients along with the release of sphingosine 1 phosphate (S1P) by pericytes at the corticomedullary junction (38, 39). S1P engages the S1P1 receptor on the mature SP thymocytes, facilitating their transit into the peripheral circulation. The hematopoietic cell seeding of the thymus and the developmental processes of positive and negative selection are maintained throughout life. However, the efficiency of these processes is dramatically curtailed during aging as adipogenesis, epithelial-to-mesenchymal transitions coupled with fibrosis disrupts the 3D structure of them meshwork and antagonizes thymopoiesis.

**Table 1:** Ligand/Receptor interactions supporting T cell development.

Ligand (Cell sources)	Receptor (Cell types)	Functional role in the thymus
BMP4 (Mes and TECs)	BMPR1/2 (Endothelial cells, TECs and Mes)	Thymus specification, TEC growth
VEGF (Mes, immature TECs and thymocytes)	VEGFR (endothelial cells)	Vascular and perivascular formation, maturation of thymic epithelium,
FGF7/10 (Mes)	FGFR2IIIb (TECs)	Growth of TECs
IGF1/2 (Mes)	IGFR 1 (TECs, thymocyte progenitor cells)	TEC expansion, thymus progenitor cell growth
EPH (Mes, TECs)	EPHR (Mes/TECs)	Descent of thymus lobes into mediastinum
S1P (Mes-pericytes)	S1PR (ETPs, mature thymocytes)	Entry of EPTs, egress of SP thymocytes
Retinoic Acid (RA) (Mes)	RAR (TECs)	Supports TEC development
lymphotoxin and LIGHT (SP thymocytes	LTbetaR (endothelial cells) LTbetaR (mTEC subset)	Regulate thymocyte homing SP thymocyte entry into the medulla
FLT3 (TECs)	FLT3 receptor = CD135 (ETPs)	Growth of early thymus progenitors
CCL25 (TECs)	CCR9 (ETPs, hematopoietic cells)	Recruitment of thymus progenitor cells
CXCL12 (cTECs)	CXCR4 (ETPs, thymocyte subsets)	ETP localization, thymocyte trafficking
CCL19, CCL21, CCL2 (TECs)	Various (thymocyte subsets) CCR7 (SP thymocytes)	Thymocyte trafficking
DLL1 (cTECs and mTECs)	Notch1 (ETPs)	Human γδ T-cell development, αβ T-cell specification in mice
DLL4 (Mes, endothelial cells, and TECs)	Notch1 (ETPs)	T-cell commitment and differentiation of thymus-seeding progenitors
JAG1 (TECs)	Notch1 (ETPs)	Controls the critical lymphoid versus myeloid developmental choice in the human thymus
JAG2 (cTECs, CMJ)	Notch (ETPs)	crucial for human γδ T-cell development, but impairs αβ T-cell development
IL-7 (TECs)	IL7Ra/IL2Rg chain (DN thymocytes)	Growth and expansion of immature thymocytes
IL-15 (mTECs)	IL15R	iNKT1 and γδT1 cell development.
CD40 (mTECs, thymic dendritic cells)	CD40L (CD4+ SP thymocytes)	Required for SP T cell selection and T reg development
VCAM-1 (cTECs at CMJ)	VLA4 (Immature DN)	Selection of immature DN
MADCAM1 (Neonatal endothelial cells)	CD44 (fetal ETPs)	Migration of T lymphocyte progenitors
EGF (SP thymocytes)	EGFR (mTECs)	Modulates fetal thymocyte growth and differentiation
FGF21 (cTECs)	bKlotho (TECs; Endo at CMJ)	Limits adipogenesis
IL-23 (thymic DC, ILCs type 11)	IL12bR-IL-23R (Mature DP thymocytes)	Negative selection of CD4^hi^CD8^hi^
IL-22 (thymic DCs)	IL22R1-IL10R2 (TECs)	Cell survival
PDGFa/c (TECs)	PDGFR (Mes)	Mes proliferation, survival and movement
RANK (ILC3)	RANKL mTEC(II)s /lymphatic endothelial cells	Optimal differentiation of mTECs
CD34 (Mes)	SELL (fetal ETPs)	Migration of T lymphocyte progenitors and precursors

### Neural Crest-Derived Mesenchymal Cell and Endothelial Cell Contributions to the Thymic Structure and Microenvironment

Neural crest cell (NCC)-derived mesenchymal cells (Mes) and endothelial cells are two key cell types essential for thymus formation, establishing the thymus vasculature. The vasculature serves as the entry site for T lymphocyte precursors or early thymus progenitors (ETPs), generated in fetal liver and bone marrow, to the thymic cortex. Endothelial cells form a cushion around the developing arterioles and veins, with the perivascular space comprising collagen fibers. Perivascular cells include pericytes and vascular smooth muscle cells (VSMCs) (40). The thymus anlage forms as Mes first localize around the 3^rd^ PP (23). Mes release BMP4 and Sonic Hedgehog (SHH) in a spatially and temporally defined manner, initiating the patterning of the thymus and parathyroid regions, respectively (1, 7). Fibroblast growth factors (FGF7 and FGF10) and insulin growth factors (IGF1 and IGF2), secreted by Mes, are bound by the corresponding FGF- (FGFR2IIIb) and IGF- receptors (IGFR1) expressed on immature TECs, providing growth signals to the latter (41, 42). Multiple FGFs (FGF3, FGF8, FGF10, FGF15) are differentially expressed in a regionalized manner (43). Dysregulated activation of the FGF pathway leads to thymus hypoplasia, revealing the importance of temporal control of FGF expression levels (42, 43). A key role for Mes in thymus development has been shown using several distinct experimental approaches. First, extirpation of the cephalic neural folds in chick embryos (stage 9), a technique that depletes NCC-mesenchymal cells, results in thymus hypoplasia/aplasia (44). Second, mechanical removal of the Mes capsule from murine e12.5 thymuses causes a stunted expansion of the lobes when placed in culture (41, 45). Extraction of the capsule also limits tissue expansion when the thymus is grafted under the adult kidney capsule, despite adult kidney mesenchyme surrounding the tissue (41, 46–48). In the capsule stripped thymuses, T cell development is normal, as only a reduced cell number is noted relative to controls (41). Epidermal growth factor (EGF) addition can replace thymic mesenchyme to induce the lobulation of e13 embryonic thymuses, however this can occur in the absence of thymocytes (49, 50). Third, 22q11.2 deletion syndrome (often termed DiGeorge syndrome) causes congenital hypoplasia of the thymus (51, 52). The primary reason is a failure of Mes to support tissue expansion, established using murine models of the syndrome. Mesenchymal cells also induce MHC class II molecules on epithelial cells (49).

As the two thymus lobes expand, they pair and descend along the right and left subclavian arteries. The descent (between e11.0 and e12.5) involves erythropoietin-producing hepatocellular carcinoma (EPH) ligand-EPH receptor (EPHR) coupled Mes and TEC signaling (53). The Mes cells that differentiate into pericytes during embryogenesis are retained post-birth and into adulthood, determined by fate mapping neural crest cells with an embryonic specific Sox10-driven Cre expression (23, 54, 55). Such pericytes secrete sphingosine 1 phosphate (S1P) at the corticomedullary junction, which is needed to recruit thymus progenitor cells and enable the egress of the mature thymocytes into the circulation (38). Most studies have grouped the Mes populations collectively, principally based on the expression of Pdgfrα and/or fate mapping. More recent deep sequencing technologies, particularly single cell RNA sequencing is revealing a more complex heterogeneity among Mes cells (56, 57). Five Mes subtypes are evident in the developing embryonic thymus (**Table 2**), among these are pericytes, vascular smooth muscle cells and fibroblasts. Recent studies have revealed some heterogeneity between the capsular and medullary fibroblasts (58). Comparing human thymuses from fetal stages as well as postnatally by scRNA sequencing reveal 3 Mes subtypes; Fibroblast type 1 (*PDGFRA*, *COLEC11*, *C7*, *GDF10*), Fibroblast type 2 (*PDGFRA*, *PI16*, *FN1*, *FBN1*) and VSMCs (*ACTA2*) (**Table 2**) (57). Fibroblast types 1 and 2 (Fb1 and Fb2) are localized in peri lobular or interlobular positions, respectively. Fb1 cells express genes such as Aldehyde dehydrogenase 1A2 (*ALDH1A2*), which is needed for retinoic acid (RA) production, a morphogen that supports TEC development (47). Fb2 are often associated with large blood vessels lined with VSMCs. Fb2 express extracellular matrix protein and semaphorins that aid in vascular development (57). Notably, the fibroblast composition changes over time, with a Fibroblasts type 1 (Fb1) population prevalent in early developmental stages, while type 2 (Fb2) dominates in post-natal and adult thymus tissue (57). The antigens produced by these fibroblast subsets have a key role in central tolerance (58).

**Table 2:** Distinct cell types in the thymus identified with single cell RNA sequencing.

Embryonic murine thymus		Human thymus stroma		Fetal/Adult thymus		Human embryonic thymus	
Cell designation	Key gene identifiers	Cell designation	Key gene identifier	Cell designation	Key gene identifier	Cell designation	Key gene identifier
M-1 Fibroblasts type 1 (Fb1)	Pdgfra, Gdf10, Aldh1a2, Col1a2, Col3a1, Sfrp2, Ntrk2	Mesenchymal	PDGFRA, LUM, LAMA2	Fibroblasts type 1 (Fb1)	PDGFRA, COLEC11 C7, GDF10, ALDH1A2	Mesenchymal Supercluster	PDGFRA, PDGFRB COL1A2, COL1A1, COL3A1, NTRK2, LUM, MEST, DCN, DLK1, PTN
M-2	Pdgfra, Mest, Lum, Gdf10, Col1a2, Dlk1, Dcn11			Fibroblasts type 2 (Fb2)	PDGFRA PI16, FN1, FBN1		
M-3	Pdgfra, Col1a2, Col3a1, Mest, Lum						
M-4	Col1a2, Itm2a, Mgp, Vim	Lymphatic endothelial	LYVE1, PROX1, CCL21	Endothelial	CDH5 PECAM1, LYVE1		
M-5 Pericytes, Vascular smooth muscle, Fibroblasts 2	Pdgfrb Acta2 Rgs5, Mcam, Cspg4, Fbn1, Fn1	Pericyte	PDGFRB, MCAM, CSPG4	VSMC	ACTA2, PDGFRB RGS5		
Endothelial-1	Cdh5 Pecam1, Cav1, Plvap, Cldn5, Esam	Vascular arterial endothelial	PECAM1, VEGFC, GJA4	Endothelial	CDH5 PECAM1, LYVE1	Endothelial Supercluster	CDH5, PECAM1 CAV1/2, CLDN5, SPP1, PLVAP, MADCAM1, TMEM88, CRP2, ESAM
E-1 cTEC^lo^	EpCAM, Krt8, Psmb11, Prss16 Ccl25	cTEC^lo^	EpCAM, KRT8 PSMB11^lo^, PRSS16^lo^, CCL2^lo^ MHC^lo^	cTECs	EpCAM, FOXN1 PSMB11	Epithelial supercluster	EpCAM, KRT8, KRT19, KRT17, KRT5, CCL25, PSMB11, PAX1, SIX1, S100A14, PRSS16
E-2 Immature TECs	Krt8, Pax1, Krt18	Immature TEC	EpCAM, KRT8 FOXN1, PAX9, SIX1				
E-3 cTEC^hi^	EpCAM, Krt8, Psmb11, Prss16 Ccl25	cTEC^hi^	EpCAM, KRT8 PSMB11, PRSS16, CCL25				
E-4 Immature TECS	EpCAM, Krt5, Krt8, Krt17, Krt19, Pax1, Six1						
E-5	EpCAM, Pth, Chga, Ccl21a, Spp1						
E-6	EpCAM, Nkx2.1, Pax8, Hhex						
		mTEC^lo^	EpCAM, KRT8 CLDN4, HLA class II^lo^, CCL21	mTEC (III)	EpCAM, KRT1		
				mTEC (I)	EpCAM, KRT14		
		mTEC^hi^	EpCAM, KRT8 SPIB, AIRE, FEZF2, HLA class II^hi^	mTEC (II)	EpCAM, FOXN1, KRT14, AIRE		
				mcTECs	EpCAM, FOXN1 PSMB11, DLK2, KRT14		
		Comeo like mTEC	EpCAM, KRT8, KRT1, IVL	mTEC (IV)	EpCAM FOXI1		
		Neuroendocrine	EpCAM, KRT8, BEX1, NEUROD1	TEC (neuro)	EpCAM, NEUROD1, CHGA		
		Myoid	EpCAM, MYOD1, KRT8, DES	TEC (myoid)	EpCAM, MYOD1 CHRNA1		
		Myelin+	EpCAM ,KRT8 SOX10, MPZ				
				mTEC (IV)	DCLK1 or POU2F3		
Hematopoietic	Ptprc, CD7, Lck, CD3d, CD52	Immune cells	PTPRC, CD3D, CD7	Immune cells	*PTPRC,NKG7, IFNG, TBX21*	Hematopoietic Supercluster	PTPRC, CD7, LCK, CD1B, CD3D, TRBC2, CD3G, PTCRA, CD52
Red blood cells	Hba, Hbb	Red blood cells	GYPA, HBA1, HBG1				

Endothelial cells are a second, critical non-hematopoietic cell type, needed for effective thymopoiesis. ScRNA sequencing data suggests the existence of one major endothelial cell type (56, 57, 59). As described in an earlier section, these cells form the vasculature/blood vessel network in the thymus. Of critical relevance to the specification of the embryonic thymus, some endothelial cells differentiate into TECs. The scRNA sequencing studies coupled with bioinformatics screens for paired ligand-receptor interactions between endothelial cells and thymocyte progenitors also reveals important contributions of MADCAM1-CD44, CFH-SELL, and CD34-SELL in thymopoiesis **(** **Table 1** **)**. In adult tissues, endothelial cells regulate thymocyte homing *via* the lymphotoxin β receptor (LTβR) (60). The ligands for LTβR, lymphotoxin and LIGHT, are produced by mature T cells to activate endothelial-regulated homing functions. In adult thymus tissues following radiation-induced damage, the endothelial cells secrete BMP4 to support tissue regeneration (61). This is partly due to BMP4 enhancement of *FOXN1* expression (61). It is likely that the embryonic endothelial cells also produce BMP4 in developing thymuses. In the post-natal thymus, the endothelial cells in the cortical region express high levels of claudin-5, which limits cell entry. This contrasts the endothelial cells in the cortical medullary junction (CMJ) and medulla, where many of the cells have lost claudin-5 expression, enabling T cell migration and egress following positive selection (62).

### Epithelial Cell Control of Thymopoiesis

TECs are derived from endothelial cells around e10.5, beginning with the formation of immature bi-potent thymus epithelial cell (TEC) precursors (63, 64). These are defined by the expression of Cytokeratin 8, Cytokeratin 14, beta5t (encoded by *Psmb11*) and PLET1 (65). Immature TEC growth is sustained by various growth factors secreted by Mes, including FGFs and IGFs, as described above (**Table 1**). As the thymus and parathyroids coordinately expand, the immature TECs express high levels of E-cadherin, which facilitates the separation of the two tissues (53). The first identification of TEC progenitors revealed that mTECs and cTECs share a common origin (66, 67). Later complementary studies reported that embryonic TEPs expressing cortical markers can generate both cTECs and mTECs (68–70). The essential functional roles of TECs are mediated by *FOXN1* (12, 71). *FOXN1* is expressed in TECs following the initial specification of the thymus (10). The importance of *FOXN1* in TECs was best revealed with spontaneously arising murine *Foxn1* mutations along with the identification of humans who had autosomal recessive *FOXN1* mutations, resulting in a Nude/SCID phenotype (12, 72–77). The Nude designation arises from alopecia universalis and nail dystrophy (75). In the skin, loss of *FOXN1* prevents sufficient expression and deposition of keratins along the hair shaft, causing the hair follicle to curl and break instead of extruding through the epidermal layers (78). SCID arises because of an inability of the TECs to differentiate and expand without *FOXN1*, causing a block in T cell development at the early DN stage of thymopoiesis [reviewed in (13, 14, 79, 80)]. A key advance in understanding how *FOXN1* controls TEC functions was the identification of its transcriptional targets by chromatin immunoprecipitation coupled with DNA sequencing. Using a FLAG-tagged *Foxn1* BAC transgenic mouse line, a consensus nucleotide binding site of GACGC was identified (14). Among the ~500 or so direct targets of murine Foxn1 are chemokines and Notch ligands, *Ccl25*, *Cxc112* and *DLL4*, respectively (14, 81). Other important gene targets include peptidases (e.g., *Tasp1*), proteases (e.g., *Prss16*), proteasome complex components (e.g., *Psmb4*, *Psmb9*, *Psmb10*, *Psmb11*, *Psmb16*, *Psma4*), peptide transporters required for MHC class I peptide presentation (e.g., *Tap2*), many *keratins* and *Cd83* (14, 78, 81). *Psmb11* encodes beta5t, a catalytic subunit of the thymus-specific proteasome (thymoproteome), generating peptides that are bound by MHC class I to support the positive selection of CD8^+^ T cells (81, 82). CD83 is required for the development of most CD4^+^ T cells (83). *FOXN1* positively regulates itself, binding to the GACGC sequence present in its own promoter (78, 84, 85). In the skin, the Hoxc13 transcription factor positively regulates *Foxn1* expression (78). Mutations in human *HOXC13* diminish *FOXN1* levels in the skin, causing ectodermal dysplasia, characterized by atrichia and nail dystrophy (86). Several Wnt glycoproteins also positively regulate *Foxn1* expression (87). An analysis of conserved nucleotide sequences close to the Foxn1 target sequence reveals enrichment of TAp63 (one of two *p63* gene transcription isoforms) and CREB binding sites. Notably, TAp63 participates in TEC homeostasis (88–90).

As immature TECs expand, they develop into two major subsets, designated as cortical or medullary TECs based on their location within the thymus. Cortical TECs are defined by the expression of Cathepsin L, TSPP and PSMB11 [reviewed in (91)]. These cells support the recruitment of early thymus progenitors from the blood *via* selected chemokines (CCL21, CCL25) as well as the progression of thymocytes from the DN to DP stages of thymopoiesis (CCL19, CCL25, CXCL12). CCL9, CCL21, CCL25, CCL12, positively regulated by FOXN1, provide directional cues for developing thymocytes (39, 92). DLL4 levels, which are much higher on cTECs than mTECs, signal immature thymocytes *via* Notch to progress from the DN to DP subset (25, 93). The conditional targeting of DLL4 in TECs leads to a severe failure of T cell development at the DN1 stage of early DN thymocyte progression. Likewise, the elimination of the receptor (Notch 1) for DLL4 on hematopoietic progenitor cells prevents T cell development (94). Cortical TECs also secrete IL7, a cytokine that stimulates DN thymocyte growth *via* the IL7Ra/IL2Rg receptor (24, 26).

The medullary regions of the thymus form during embryogenesis as small pockets that expand and coalesce into the larger clusters present post-natally. These mTECs differentiate/proliferate following interactions with mature SP thymocytes. In the embryonic period, Notch signaling is needed for the formation of mTECs, with RANK-mediated signaling more important for the mTECs post-natally (95, 96). The differentiation involves CD4 SP thymocyte expression of Notch and/or RANK in combination with CD40L and EGFR (95–101). Once formed, mTECs are defined by the expression of Cathepsin S, CD40, CCR7 ligand (CCL19/CCL21) and AIRE [reviewed in (91)]. Medullary TECs mediate negative selection of SP T cells along with the positive selection of T regulatory cell subsets (102, 103) [reviewed in (30, 32)]. One of the mTEC subsets produces the chemokines CCL19 and CCL25 to attract positively selected, CCR7 expressing thymocytes into the medullary region (104). Mice lacking the lymphotoxin beta receptor fail to develop these mTECs (105). As the SP thymocytes traffic through the medullary region, they interact with a second subset of mTECs, defined by the expression of the autoimmune regulator (*AIRE*) gene. AIRE enforces the expression of “tissue-restricted” proteins that eliminate autoreactive T cells (33, 106). AIRE facilitates this by releasing RNA polymerase II from promoter regions where this enzyme is stalled, a mechanism normally used to prevents widespread gene expression (107). Mesenchymal epithelial transition factor (c-Met) is expressed by TECs along with early T progenitors. The specific targeting of c-Met in TECs results in age-dependent progressive reduction in TECs number coupled with lower regulatory T cells (108). Similarly, the targeted loss of Shh in TECs reduces both cTEC and mTEC numbers (109). While most studies have detailed the development of cTEC and mTEC from embryonic TEC progenitors, thymus epithelial progenitors have also been identified in adult tissues (110, 111). These adult progenitor populations can give rise to both cortical and medullary TEC subsets (110, 111).

While initial characterizations of TECs relied on cell surface protein expression and fate mapping to define subsets, scRNA sequencing is revealing many additional epithelial and TEC subsets. For example, an analysis of embryonic day 13-13.5 fetal thymuses, when 80% of the cells are stromal (Mes, TEC, endothelial), reveal 6 distinct epithelial subsets (**Table 2**). Multiple other studies have revealed different TEC populations at different developmental stages (112–116). Among these are 2 subsets of immature TECs along with cTEC^lo^ and cTEC^hi^ subsets, with the lo and hi referring to MHC class II levels. At e13.-13.5 stage, no mTECs are evident. A 5^th^ epithelial subset expresses parathyroid hormone (PTH), suggesting either the presence of some parathyroid TECs or the existence of a bi-functional TEC. The 6^th^ epithelial subset (E-6) expresses *Nkx2.1*, which may be a thyroid or parathyroid precursor cell type. ScRNA sequencing of human thymuses obtained at gestational weeks 19 and 23 (e14.5-e16.5 in murine embryos) and postnatal samples from 6-day old newborn (e20) and an infant (10 months) reveals 3 major epithelial groups broken down into 9 subclusters (57). Two clusters are categorized as cTECs based on the characteristic genes (*PSMB11*, *PRSS16*, *CCL25*). One cluster is the cTEC^lo^, which is rapidly proliferating based on Ki67^+^ expression. The second cluster is the cTEC^hi^. mTECs are also evident in the embryonic tissues at this developmental stage, and split into 3 distinct subgroups partly defined by the levels of HLA class II. These are mTEC^lo^ (*CLDN4* and lower levels of HLA class II), mTEC^hi^ (*SPIB*, *AIRE*, *FEZF2*, higher levels of HLA class II), and corneocyte-like mTECs (*KRT1*, *IVL*) (56). Cells in the mTEC^lo^ cluster express high levels of the chemokine *CCL21*, similar to the CCL21-expressing post-natal mTEC^lo^ population described in mice (105). Another epithelial cluster, marked by *FOXN1*, *PAX9*, and *SIX1*, represents immature TECs, and is evident in early developmental stages and remains in postnatal and adult periods. *PAX9*, and *SIX1* are not present in cTECs or mTECs, implicating this cluster as a potential progenitor cell not yet committed to a specific lineage. The additional epithelial clusters identified in human thymuses include neuroendocrine (*BEX1*, *NEUROD1*), muscle-like myoid (*MYOD1*, *DES*), and myelin^+^ epithelial cell markers (*SOX10*, *MPZ*) (56, 57). A mixed medullary/cortical TEC subset (mcTECs), marked by expression of DLK2, is present in late fetal and post-natal human thymuses. Finally, a rare population of tuft-like mTECs is present in human and mouse thymuses, although the genes *DCLK1* and *POU2F3* used to define this population are not specific to TECs in humans (57).

The various TEC subsets constitute the basic thymic microenvironment. They provide cytokines, chemokines, multiple molecular signals, and cell-to-cell interactions to control thymocyte proliferation, differentiation, and selection. This process leads thymocytes from immature to mature, and in turn, TECs themselves also get maturation from bi-potent epithelial progenitors to functional cTECs and mTECs. The added complexity of many different subsets suggests distinct roles for each in the formation and regeneration of the thymus at different stages.

### The Changing Cellular Landscape in an Aging Thymus

Post-adolescence, the thymus begins a slow and continuous atrophy (117, 118). Tissue changes include TEC loses, increasing epithelial-to-mesenchymal transitions (EMT) coupled with their differentiation into fibroblasts, adipogenesis, loss of cortical-medullary boundaries, and increases in perivascular spaces (118, 119). Although recruitment of thymus progenitors from the bone marrow is not critically impacted, alternations in T cell development, defects in T cell selection, a contracted TCR repertoire diversity, and reduced egress of naive SP T cells are apparent with aging (120). TEC loses by both EMT and cell death, coupled with the tapered expression of *FOXN1* contribute to diminished thymopoiesis (91, 121, 122). In fact, enforced expression of *Foxn1* in an aged thymus can restore thymus size and functionality, comparable to that seen with a young thymus (123–125). As the thymus ages, TEC production of IL-7 and FGF21 tapers. FGF21 losses increase intra-thymic adipogenesis and decrease peri-thymic brown adipose tissue (126). The obligate co-receptor for FGF21 is beta-Klotho (KLB), present on the endothelial cells at the corticomedullary junction (126). Reduced signaling *via* FGF21/KLB comprises endothelial functions. However, KLB-deficient mice have a pronounced thymus hypoplasia not intrinsic to TECs or bone marrow cells, revealing a systemic effect (127). Notably, the KLB-deficient mice have high levels of vitamin D, and thymus hypoplasia is rectified by nutritional restriction of Vitamin D (127).

By mid-age, ectopic adipocytes make up 50% of the thymus (119). Although the adipocytes come from a variety of tissues, mesenchymal transitions *via* EMT are one proposed source (126). The adipocytes fill the perivascular space and impede entry of thymus progenitor cells (128, 129). Intra-thymically distributed adipocytes also release cytokines, steroids, and hormones that negatively impact thymopoiesis (130–133). Among these are leukemia inhibitory factor (LIF), oncostatin M and IL-6. In mouse models of aging, caloric restriction or systemic administration of Ghrelin improves T cell output by reducing adipogenesis and delaying age-dependent thymic involution (134, 135). EMT further generates an expanding number of fibroblasts within the thymus, increasing fibroblast/TEC ratios (136). Whether these fibroblasts directly contribute to reduced thymus functions, as seen with pulmonary fibrosis, remains to be established (137).

### Thymus Regeneration Technologies for the Young and the Old

The thymus is extremely stress sensitive, with transient cell losses reaching 90% evident following infections, glucocorticoid treatments, chemotherapy, and radiation exposure (138, 139). Dependent on the severity of the stress and/or the age of the thymus, the damage can be transient (if the damage is only in hematopoietic lineage cells) or permanent (the damage mostly happens in non-hematopoietic lineage cells). Because of this, many clinical interventions are being considered to improve/rejuvenate thymus functions. Among these are cytokines and growth factors that support the non-hematopoietic lineage stromal cell populations and/or developing thymocytes. For example, TEC differentiation/expansion can be improved with IL-22, Keratin growth factor (KGF), EGF, BMP4, RANKL and 2 microRNAs, miR-205 or miR-29a (61, 97, 99, 140–144). Group 3 innate lymphoid (ILC3s) cells in the thymus are one important cell population that facilitate thymus recovery following stress (145). In response to whole body radiation exposure, CD103^+^ thymus dendritic cells release IL-23, which signals the ILC3s to secrete IL-22. The IL-22 receptor (IL-22Ra/IL-10Rb) is principally expressed on cTECs and mTECs, with ligand mediated signaling supporting TEC survival and functionality (141). BMP4, produced by endothelial cells, similarly enhances TEC functions, including an upregulation of *FOXN1* along with its downstream targets (61). Additional cytokines, ligands and chemokines that improve T cell development include IL-7, Fms-Like Tyrosine Kinase 3 Ligand (FLT3L), DLL4 and the chemokines CXCL12, CCL19, CCL21, and CCL25 (91, 146–150). In a clinical setting, enhanced thymopoiesis is accomplished by administration of FLT3L, and this is done for patients receiving a bone marrow transplant (151–153). FLT3L increases progenitor cell uptake into the thymus (152). The potential use of the many of the other proteins that support thymopoiesis for clinical treatments requires further validation as many will impact cell populations outside the thymus. Two possible solutions to overcome the pleotropic effects of these various cytokines/chemokines or ligands include direct intrathymic injections, or administration of encapsulated nanoparticles with selectively for the thymus (154, 155). For example, intrathymic injections of Foxn1, consisting of an COOH-terminal TAT transduction domain linked to full-length Foxn1, transiently improves TEC numbers and thymopoiesis in mouse models of hematopoietic stem cell transplantation (155). However, recombinant Foxn1 protein injections into a thymus also result in the protein entering hematopoietic cells, and this can have potentially harmful outcomes (156). Therefore, direct intrathymic injections of post-natally derived TECs or with Foxn1-reprogrammed fibroblasts are alternate strategies with good rejuvenation effects (157, 158). Furthermore, direct intrathymic injections of encapsulated cytokines and/or growth factors and even reprogrammed cells into the thymus may be of therapeutic value.

For individuals with in-born errors of immunity, the clinical treatment options for restoring thymopoiesis are not straightforward. First, an assessment of whether hematopoietic or stromal cell populations are causal to thymus hypoplasia is needed. If genetic mutations are inherent to the hematopoietic cells, bone marrow transplants are an effective clinical treatment (159). However, individuals wherein the non-hematopoietic stromal/epithelial cells of the thymus are impacted, allogeneic thymus tissue transplants remain the only FDA approved therapy (Enzyvant, Inc.) (160–162). For such tissue transplants, the donor thymus is depleted of most hematopoietic cells, leaving a residual mixture of pericytes, endothelial cells, TECs, and some mature CD4^+^ T cells (160). These transplant strategies work for those with a severe T cell lymphopenia. Among the patients who will benefit from such transplants include those with 22q11.2 deletion syndrome (DiGeorge), autosomal recessive *FOXN1* mutations, *PAX1* mutations, CHARGE (Coloboma, heart defects, anal atresia, growth retardation, genital abnormalities, ear abnormalities) syndrome, or those who had diabetic embryopathies (6, 80, 160). However, the allogeneic transplant approach is not suitable for individuals who retain some thymus functionality, since they will have sufficient peripheral T cell numbers to cause transplant rejection. Among these are patients undergoing chemo ablative therapies. An equally important group to consider are those who had partial or complete thymectomies, often done during restorative cardiothoracic surgeries since the thymus affects surgical access to the heart (163–165). In individuals who were thymectomized at a young age, low naïve peripheral T cell numbers and a reduced TCR repertoire is evident later in life (166, 167). Noteworthy, complete thymectomies are also done for patients with thymomas or the autoimmune disorder Myasthenia gravis (168). Sadly, radiation-mediated thymus ablation was a common standard-of-care treatment for infants during the years 1910-1960 (169, 170). The prevailing medical notion at the time was that a large thymus (which is actually normal) was causing lung compression and/or asthma, leading to sudden infant death syndrome and status thymicolymphaticus (171). Regardless of reasons for why a thymus is “removed”, there are currently no effective strategies for restoring thymopoiesis. Putative solutions emerge from recent advances in thymus regeneration technologies, supported with two lines of evidence. First, it is clear thymus transplants work for patients with a thymus aplasia, as well as with the Nude/SCID mice, where the transplanted tissue supports T cell development (161, 162, 172, 173). This indicates that artificial thymic organoid (ATO) technologies may become an effective clinical approach for tissue regeneration. Second, organoid technologies are rapidly advancing for many types of tissues, although regenerating an effective thymus remains a significant challenge (91).

A quintessential breakthrough for ATOs was the initial development of stromal cell lines (OP9) expressing the Notch ligands Delta-like ligand 1 (DLL1) or DLL4, with both supporting T cell development to the SP stage by engaging Notch on immature thymocytes (174, 175). DLL4 functions better than DLL1 due to the lack of a proline rich domain at the Module at the N-terminus of Notch Ligand (MNNL) (176). This is consistent with the non-redundant role for DLL4 *in vivo*. An important modification to the OP9 monolayer system was the use a unique murine stromal cell line that re-establishes a 3-d epithelial meshwork upon co-culture with hematopoietic stem and progenitor cells (HSPC) (177). HSPCs can be from either cord- or peripheral- blood or bone marrow (177). While the stromal cell lines were first developed to express human DLL1 (MS5-hDLL1), human DLL4 is now used (177–179). Grown in serum-free culture conditions to eliminate variations caused by serum, human T cell differentiation proceeds to the SP stage in the ATOs (177–179). While the ATO technology is a definite improvement, the organoids are not designed for transplant purposes. Limitations include the low overall numbers of T cells that are generated (1 to 3 x 10^6^ cells), an abnormal CD4/CD8 ratio < 1 and the use of murine cell lines. ATOs can, however, reveal whether a patient with probable in-born error of immunity would need a bone marrow or thymus transplant. For example, patients with mutations in genes affecting stromal cell subsets (mesenchymal, TECs, endothelial) will retain HSPCs capable of thymus development (178, 179). Those with mutations affecting either hematopoietic cell differentiation and or T cell functions (IL2Rg chain, RAG, Artemis, T cell components) will not support T cell development, confirming that a patient will likely need a bone marrow transplants. Notably, the current ATOs now use DLL4 expressing stromal cells (MS5-hDLL4) cultured with patient derived CD34^+^ cells (178, 179).

A third strategic advance for improving thymus organoid technologies is the addition of decellularized thymus scaffolds. Comprising the extracellular matrix from thymuses, these support TEC recolonization and bone marrow cell reconstitution. The reconstituted scaffolds are grafted under the kidney capsule of nude/SCID mice TEC and support T cell development (180–183). Disadvantages of such scaffold approaches include the need for primary human thymus tissues, complex experimental manipulation, and low cell yields. However, recent research has revealed techniques to expand TECs and interstitial cells from human thymuses in large numbers, potentially suitable for clinical approaches (184). Another strategy to generate many functional TECs is to use embryonic fibroblasts, which are reprogrammed with Foxn1 over-expression systems, and are termed inducible TECs. These cells can be reaggregated to generate an ectopic *de novo* thymus under the kidney capsule in the mouse models (48). Host T cell progenitors seed the *de novo* thymus-like organ generated by the transplant, and normal thymocyte distributions are observed after 4 weeks. Additionally, typical thymus microstructures are evident in the *de novo* thymus engrafted tissue (48). Combining the various techniques holds promise for personalized thymus regeneration approaches.

## Discussion

The key to effective thymus regeneration requires knowledge of how particular cell subsets contribute to thymopoiesis (**Tables 1**, **2**). Thus, ad-mixing various cell subsets from different tissues, isolated at distinct development stages, will likely be ineffective. A multi-omics profiling of key stromal cell populations; endothelium, epithelium and fibroblasts, performed with 12 distinct mouse organs including the thymus, reveals global transcriptome patterns that are quite divergent when comparing similar cell types obtained from different tissues (185). In fact, the 3 major stromal populations are more related transcriptionally when obtained from the same tissue source, such as the thymus. This suggests that individual tissue environments create transcriptional similarities among the stromal cell populations (185). Such a finding argues that selection of the “correct” stromal tissue from a “thymus” programmed environment is essential for developing effective thymus organoids. Notably, thymus organogenesis involves strategic cellular interactions among different cell clusters using selected receptor-ligand pairs (186). By integrating scRNA sequencing with known receptor-ligand pair interactions, the connectivity among TECs, mesenchymal cells, and endothelial cells is emerging (59). Combing this information with the HLA haplotype of the cells needed for the host and considerations about the patient, thymectomized versus one with an in-born error of immunity, will better inform thymus regenerative technologies. The 4 key cell types to consider for thymus regeneration are mesenchymal cells, endothelial cells, endothelial-derived TECs and hematopoietic stem cells with adequate thymopoietic potential. The last two decades of thymus research suggest that personalized thymus regeneration techniques incorporating these 4 cell types are nearing realization.

## Author Contributions

PB, D-MS, and NvO wrote the manuscript. All authors contributed to the article and approved the submitted version.

## Funding

Our work was supported, in part, by grants from the National Institutes of Health. National Institutes of Health grant R01AI114523 (NvO) National Institutes of Health grant R21AI144140 (NvO).

## Conflict of Interest

The authors declare that the research was conducted in the absence of any commercial or financial relationships that could be construed as a potential conflict of interest.

## Publisher’s Note

All claims expressed in this article are solely those of the authors and do not necessarily represent those of their affiliated organizations, or those of the publisher, the editors and the reviewers. Any product that may be evaluated in this article, or claim that may be made by its manufacturer, is not guaranteed or endorsed by the publisher.
